# Warming Diminishes the Day–Night Discrepancy in the Apparent Temperature Sensitivity of Ecosystem Respiration

**DOI:** 10.3390/plants13233321

**Published:** 2024-11-26

**Authors:** Nan Li, Guiyao Zhou, Mayank Krishna, Kaiyan Zhai, Junjiong Shao, Ruiqiang Liu, Xuhui Zhou

**Affiliations:** 1 Northeast Asia Ecosystem Carbon Sink Research Center (NACC), Key Laboratory of Sustainable Forest Ecosystem Management, Ministry of Education, School of Ecology, Northeast Forestry University, Harbin 150040, China; 2 Laboratorio de Biodiversidad y Funcionamiento Ecosistémico, Instituto de Recursos Naturales y Agrobiología de Sevilla (IRNAS), Consejo Superior de Investigaciones Científicas (CSIC), 41012 Sevilla, Spain; 3 College of Forestry and Biotechnology, Zhejiang A&F University, Hangzhou 311300, China

**Keywords:** ecosystem respiration, temperature sensitivity, diel variation, eddy covariance, FLUXNET

## Abstract

Understanding the sensitivity of ecosystem respiration (ER) to increasing temperature is crucial to predict how the terrestrial carbon sink responds to a warming climate. The temperature sensitivity of ER may vary on a diurnal basis but is poorly understood due to the paucity of observational sites documenting real ER during daytime at a global scale. Here, we used an improved flux partitioning approach to estimate the apparent temperature sensitivity of ER during the daytime (E_0,day_) and nighttime (E_0,night_) derived from multiyear observations of 189 FLUXNET sites. Our results demonstrated that E_0,night_ is significantly higher than E_0,day_ across all biomes, with significant seasonal variations in the day–night discrepancy in the temperature sensitivity of ER (ΔE_0_ = E_0,night_/E_0,day_) except for evergreen broadleaf forest and savannas. Such seasonal variations in ΔE_0_ mainly result from the effect of temperature and the seasonal amplitude of NDVI. We predict that future warming will decrease ΔE_0_ due to the reduced E_0,night_ by the end of the century in most regions. Moreover, we further find that disregarding the ΔE_0_ leads to an overestimation of annual ER by 10~80% globally. Thus, our study highlights that the divergent temperature dependencies between day- and nighttime ER should be incorporated into Earth system models to improve predictions of carbon–climate change feedback under future warming scenarios.

## 1. Introduction

Terrestrial ecosystem respiration (ER), the sum of autotrophic respiration from primary producers and heterotrophic respiration from consumers and detritivores, is the largest source of CO_2_ emission to the atmosphere. Therefore, an accurate estimation of ER is crucial for understanding the global carbon cycle and for long-term climate change projections [1,2]. Early-generation carbon cycle models approximated respiration as a temperature-sensitive process, and quantification of the temperature sensitivity of ER (Q_10_) [3] was done employing equations of Lloyd and Taylor [4] and the Arrhenius equation [5] fixing Q_10_ at a constant value. The impact of temperature on ER is guided by the temperature sensitivity of individual organisms’ physiology, community composition, and biotic interactions within the ecosystem [6,7]. However, empirical- and process-based models currently fail to accurately estimate the temporal variability of ER compared with in situ observations due to the inaccuracy in parameterizing Q_10_ [2,8].

Currently, numerous empirical and theoretical studies have explored the relationships between ER and temperature at spatial scale but report contradictory conclusions. For example, some studies have observed latitudinal variations in the temperature sensitivity of ER, with higher latitudes exhibiting greater increases in ER with increasing temperature compared to mid and low latitudes [9,10]. However, global meta-analyses suggested a convergence in the temperature sensitivity of ER across different climatic regimes and ecosystem types under ongoing climate warming [2]. In addition, climate warming may stimulate ER since plant and microbial processes are sensitive to increasing temperature. However, at a biogeographical scale, climate warming rates are highly heterogenous, in addition to warming being uneven at diurnal, seasonal, and annual scales, e.g., nighttime temperatures have increased faster than daytime temperatures [11]. Such asymmetric climate warming across different scales may have important implications on ER. It is well documented that the response of ER to temperature can vary over time [2,12,13,14], but much less is known about whether the temperature sensitivity of ER is consistent across the diurnal cycle.

Nowadays, nighttime ER is measured directly using the eddy covariance technique at the ecosystem level, while daytime ER is partitioned from the net ecosystem exchange of CO_2_ (NEE) measured during the day. Generally, ER estimates predominantly utilize exponential functions to describe the temperature response of respiration, such as the Q_10_ function [9], the equations of Lloyd and Taylor [4], or Arrhenius functions [5]. Although the effect of temperature on ER may be non-linear [12], debates have long prevailed on whether the temperature sensitivity of ER differs between day and night due to inconsistent findings across studies. For example, several studies have reported a higher nighttime temperature sensitivity of respiration in a subalpine forest [15] and a temperate forest [16] and lower nighttime temperature sensitivity of respiration in peatland [12] compared to daytime temperature, while no significant difference in the temperature sensitivity of respiration was observed between day and night in a beech forest [17] and a temperate plantation forest [18]. Meanwhile, how the response of ER to temperature varies during the day and night as well as its seasonal variation across latitudes and ecosystems remain unclear. The prevailing hypothesis is that day- and nighttime ER follows the same response to air or soil temperature in ER equations [13,14]. However, extrapolating the nighttime ER to estimate daily ER may lead to overestimation due to the asymmetric pattern of diurnal variation in soil and air temperatures and the inhibition of leaf respiration in the presence of sunlight [12,16,19].

Investigating the variations in the temperature sensitivity of ER during the day (E_0,day_) and night (E_0,night_) was earlier constrained due to the inability to know the actual ER during daytime. At the ecosystem scale, empirical models generally adopt two strategies to partition daytime net ecosystem exchange (NEE) into daytime ER and gross primary production (GPP): the nighttime method (NT) [14] and daytime method (DT) [20]. The NT method uses nighttime eddy covariance observations to estimate two parameters (i.e., the reference respiration (R_ref_) and the temperature sensitivity (E_0_)) and then calculates daytime ER, whereas the DT method applies nighttime measurements to parameterize E_0_ and daytime measurements to parameterize R_ref_. Recent advances in eddy covariance measurements of stable carbon isotopic composition potentially partition NEE into daytime ER and GPP [21,22,23], exploiting the fact that ER and GPP have ^13^C/^12^C signatures that are almost always distinguishable in sub-hourly NEE. The study found that the daytime ER is overestimated using standard empirical models. However, applying these techniques at a global scale remains a daunting task due to the paucity of sites with isotopic observations. Notably, a recent study [13] introduced an improved flux partitioning method, termed DT-RH (i.e., daytime method with air relative humidity), which produced daytime ER estimates closely aligned with those derived from isotopic data. DT-RH modeling incorporates the dependency of ER on the diurnal variations in temperature and the effect of moisture on it. The robustness of DT-RH modelling was further validated using a couple of machine learning methods [13]. Therefore, relying on this in the present study, we applied the method of [13] to the global flux dataset FLUXNET2015 to obtain the temperature sensitivity of ER (E_0_) and assessed variability in the temperature dependence of ER at diurnal and seasonal scales across nine distinct ecosystem types. We also predict changes in spatial patterns of E_0_ and ER under future climate warming scenarios. Specifically, we aim to test three hypotheses: (1) the divergent temperature dependencies of day- and nighttime ER (ΔE_0_) exist in all biomes; (2) the magnitude of ΔE_0_ varies across time and biomes; (3) and the temperature sensitivities of ER during the day and night may converge under future climate warming.

## 2. Results

### 2.1. Diel Variation in the Temperature Sensitivity of ER

**Figure 1:**
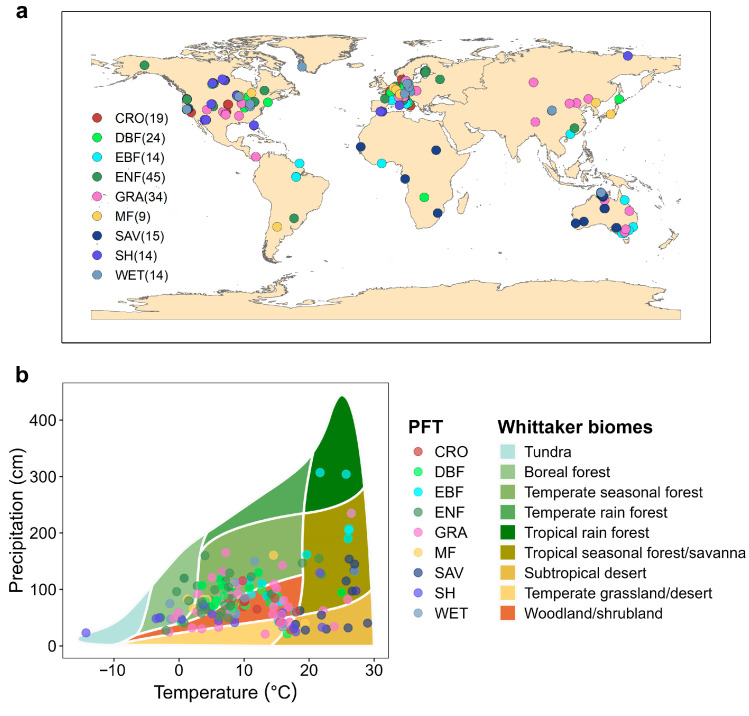
Distribution of 189 flux sites from FLUXNET 2015. (**a**) Location of each site on the latitude and longitude grid; (**b**) Location of each site according to the classic Whittaker Biome Classification based on the climate variables of mean annual temperature and precipitation. CRO, cropland; DBF, deciduous broadleaf forest; EBF, evergreen broadleaf forest; ENF, evergreen needleleaf forest; MF, mixed forest; GRA, grassland; SAV, savannas; SH, shrubland; WET, wetland.

**Figure 2:**
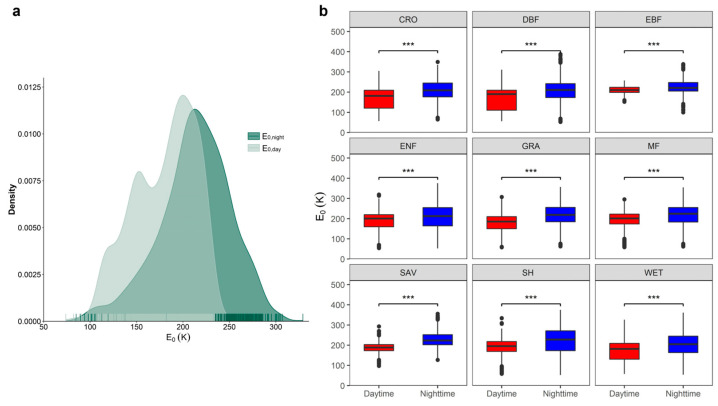
Day–night discrepancy in the temperature sensitivity of ER (E_0_). (**a**). Density plot of the daytime (E_0,day_) and nighttime (E_0,night_) temperature sensitivity of ER, which were estimated from DT-RH flux partitioning algorithms. (**b**). The difference in daytime and nighttime E_0_ among nine ecosystem types. *** *p* < 0.001; CRO, cropland; DBF, deciduous broadleaf forest; EBF, evergreen broadleaf forest; ENF, evergreen needleleaf forest; MF, mixed forest; GRA, grassland; SAV, savannas; SH, shrubland; WET, wetland.

**Figure 3:**
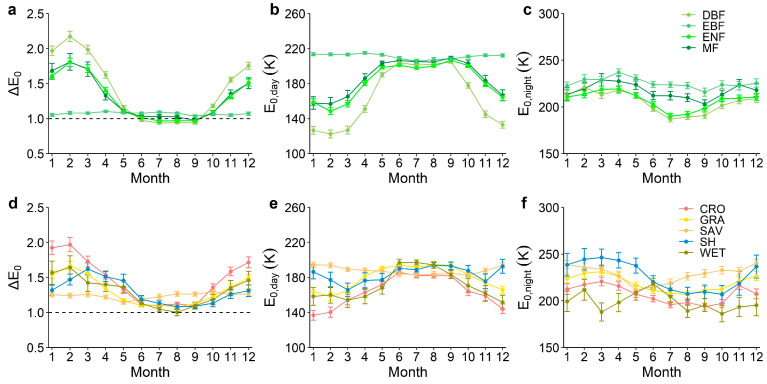
Average monthly daytime (E_0,day_) and nighttime (E_0,night_) temperature sensitivity of ER and their discrepancies (ΔE_0_ = E_0,night_/E_0,day_) among different vegetation types. (**a**–**c**) Mean monthly ΔE_0_, E_0,day_ and E_0,night_ in DBF, EBF, ENF and MF, respectively. (**d**–**f**) Mean monthly ΔE_0_, E_0,day_ and E_0,night_ in CRO, GRA, SAV, SH and WET, respectively. If ΔE_0_ > 1, it means that the E_0_ during the day is smaller than at night, and vice versa. CRO, cropland; DBF, deciduous broadleaf forest; EBF, evergreen broadleaf forest; ENF, evergreen needleleaf forest; MF, mixed forest; GRA, grassland; SAV, savannas; SH, shrubland; WET, wetland.

Using the DT-RH flux partitioning method, we estimated the temperature sensitivity of ER (E_0_) during the day and night at 189 eddy covariance flux sites distributed across the globe (Figure 1). Overall, the values of E_0,night_ were significantly higher than E_0,day_ across nine ecosystem types included in this study (*p* < 0.001, Figure 2). At the monthly timescale, the E_0,day_ values peaked from June to September for DBF, ENF, MF, CRO, GRA, SH, and WET ecosystems, whereas E_0,day_ values of EBF and SAV ecosystems had no significant variations on the monthly scale (Figure 3). Conversely, for all biomes except EBF, SAV, and WET, E_0,night_ reached the lowest value from June to September.

The day–night discrepancy in the temperature sensitivity of ER (ΔE_0_ = E_0,night_/E_0,day_) varied across ecosystems and time (Figure 3). The diurnal difference in temperature sensitivity of ER decreases between June and September in different ecosystem types, except for EBF and SAV, whereas the temperature sensitivity of ER is significantly lower during the day than at night in other months.

### 2.2. Seasonal Variations in Day–Night Discrepancies in ER Temperature Sensitivity

**Figure 4:**
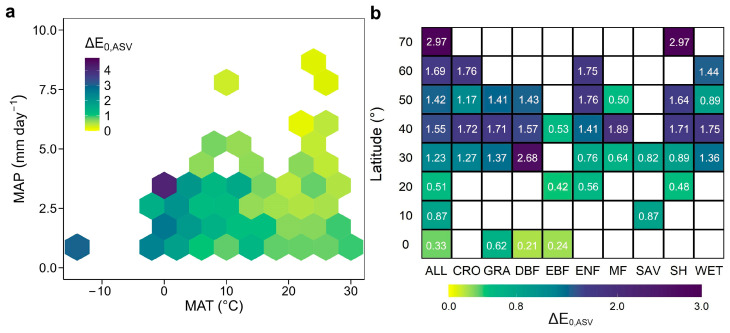
The distribution of the seasonal amplitude (ASV) in the E_0,night_/E_0,day_ ratio (ΔE_0,ASV_) across the temperature–precipitation space (**a**) and latitude (**b**). ΔE_0,ASV_ was calculated as the difference between highest and lowest monthly E_0,night_/E_0,day_ ratios from January to December. Each climatic bin is 4 °C (temperature) by 1 mm day^−1^ (precipitation) in panel a. CRO, cropland; DBF, deciduous broadleaf forest; EBF, evergreen broadleaf forest; ENF, evergreen needleleaf forest; MF, mixed forest; GRA, grassland; SAV, savannas; SH, shrubland; WET, wetland.

We used the seasonal CV and amplitude (ASV) of E_0_ to quantify its strength in seasonality. The global average seasonal CV of E_0,night_, E_0,day_, and ΔE_0_ is 19.5%, 20.3%, and 29.6%, respectively. EBF and SAV had the smallest seasonal variability in E_0_ than other biomes (Figures S1 and S2). The relationship between the seasonal amplitude in the day–night discrepancy in ER temperature sensitivity (ΔE_0,ASV_) and background climate is shown in Figure 4. Specifically, the spatial sensitivity of ΔE_0,ASV_ generally decreased with increasing mean annual temperature and precipitation (Figure 4a). In order to investigate the overall spatial patterns of ΔE_0,ASV_, we binned sites of individual biomes at latitudinal intervals of 10° and calculated averages of multi-year ΔE_0,ASV_ for each group of sites (Figure 4b). ΔE_0,ASV_ showed a pronounced increasing trend with latitudes globally. Regarding GRA, DBF, ENF, and SH, the ΔE_0,ASV_ was stronger at middle to high latitudes (≥30°) than at low latitudes (≤30°), while the increasing trend with latitudes was smaller for EBF, MF, SAV, and WET.

### 2.3. Environmental Context Modulates the Temperature Sensitivity of ER

**Figure 5:**
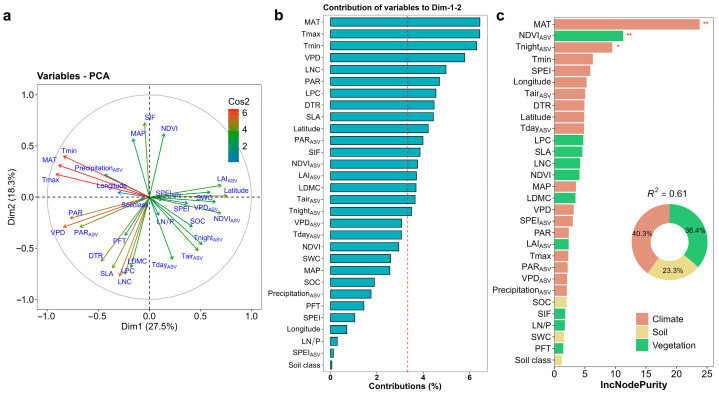
The relative contribution of environmental and plant variables to the seasonal amplitude (ASV) of E_0,night_/E_0,day_. (**a**,**b**) Principle component analysis of environmental and plant variables for calculating their relative contributions on the seasonal amplitude (ASV) of E_0,night_/E_0,day_. Contribution of environmental and plant variables to Dim 1 and Dim 2 of the principal component analysis in panel b, and the red dashed line represents the average contribution. (**c**) Results from random forest analysis, showing the relative importance of the various drivers in ASV of E_0,night_/E_0,day_. Variable importance represents the increase in mean error (computed on the out-of-bag data) across trees when a predictor is permuted. * *p* < 0.05, ** *p* < 0.01. MAT, mean annual temperature; Tair, monthly air temperature; Tnight, nighttime air temperature; Tday, daytime air temperature; Tmax, monthly maximum air temperature; Tmin, monthly minimum air temperature; DTR, diurnal temperature range; PAR, solar radiation; VPD, vapor pressure deficit; SPEI, standardized precipitation ET index; LAI, leaf area index; NDVI, normalized difference vegetation index; SIF, sun-induced chlorophyll fluorescence; LNC, leaf nitrogen concentration; LPC, leaf phosphorus concentration; LN/P, LNC/LNP; LDMC, leaf dry matter content; SLA, specific leaf area; SWC, surface volumetric soil moisture; SOC, soil organic carbon; PFT, plant functional types. Tnight_ASV_, Tair_ASV_, Tday_ASV_, SPEI_ASV_, LAI_ASV_, PAR_ASV_, VPD_ASV_, Precipitation_ASV_, and NDVI_ASV_ are the seasonal amplitude of Tnight, Tair, Tday, SPEI, LAI, PAR, VPD, precipitation, and NDVI, respectively, from January to December.

To quantify the drivers of the seasonal variations in the day–night discrepancy in ER temperature sensitivity, we calculated the response of ΔE_0,ASV_ to various environmental factors and plant traits using the principal component analysis (PCA) and general linear models. PCA showed that the first principal component (Dim 1) and its combination with Dim 2 had significant effects on ΔE_0,ASV_ (Table 1). The best model (AICc = 359.15) of ΔE_0,ASV_ was the model that included Dim 1 and Dim 2, explaining 38% of the seasonal variation in ΔE_0_. Variables related to climate factors had the largest loading weight in Dim 1 and Dim 2 (Figure 5a,b). Meanwhile, the key role of mean annual temperature (MAT) as driver of ΔE_0,ASV_ was further established by independent random forest analyses (Figure 5c), followed by seasonal amplitude of NDVI. Our analyses indicate that the climate factors (particularly temperature) are more important drivers of ΔE_0_ variations than vegetation and soil factors, explaining 40.3% of the seasonal variation in ΔE_0,ASV_.

### 2.4. Global Estimations of ER and Its Temperature Sensitivity

**Figure 6:**
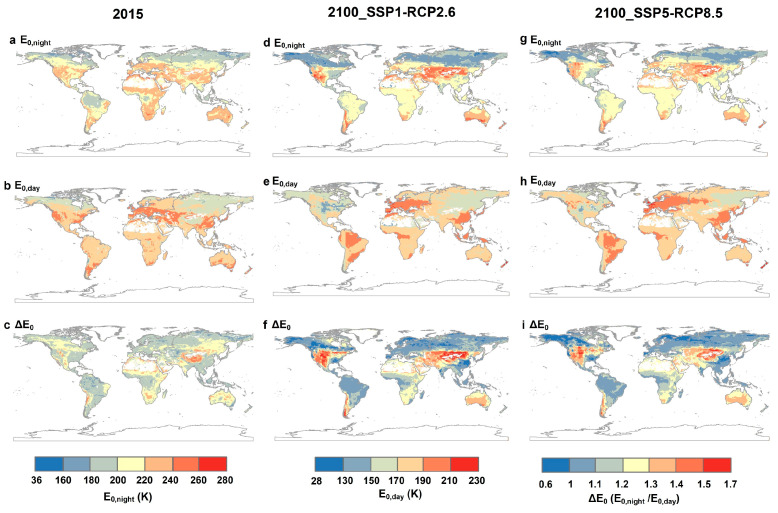
Global patterns of the daytime (E_0,day_) and nighttime (E_0,night_) temperature sensitivity of ER and their discrepancies (ΔE_0_ = E_0,night_/E_0,day_) based on historical data and future projections. (**a**–**c**) The figures shows the global patterns of E_0,night_, E_0,day_, and ΔE_0_ in 2015. (**d**–**i**) The figures shows the global patterns of E_0,night_, E_0,day_, and ΔE_0_ in 2100 under two future corresponding shared socioeconomic pathway-representative concentration pathway (SSP-RCP) scenarios (**d**–**f**, SSP1-RCP2.6; **g**–**i**, SSP5-RCP8.5).

**Figure 7:**
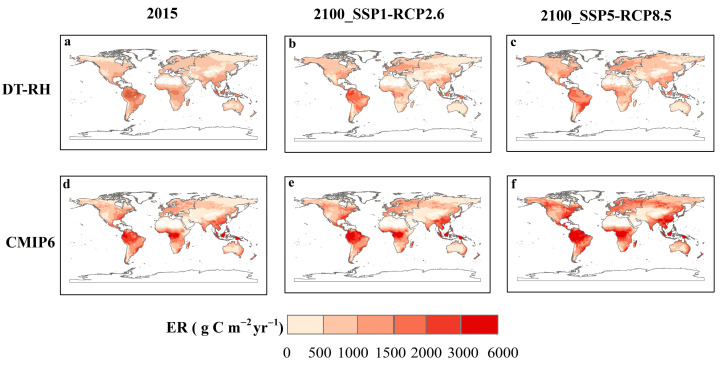
Historical and future ER values estimated from DT-RH and CMIP6 models. (**a**,**d**) The figures show the global patterns of ER in 2015 derived from DT-RH and CMIP6 models, respectively. (**b**,**c**,**e**,**f**) The figures show the global patterns of ER in 2100 derived from DT-RH and CMIP6 models under two future corresponding shared socioeconomic pathway-representative concentration pathway (SSP-RCP) scenarios.

The random forest model shows that the temporal variation in nighttime temperature is the most important factor for E_0,night_, whereas NDVI, daytime temperature, and LAI play key roles in the temporal variation in E_0,day_ (Figure S3). We further estimate E_0,day_ and E_0,night_ at 2015 and for the end of the century (2100) under two future corresponding shared socioeconomic pathway-representative concentration pathway (SSP-RCP) scenarios (i.e., SSP1-RCP2.6 and SSP5-RCP8.5) at the global scale using the random forest model. The temperature sensitivity of ER during nighttime decreases globally by the end of the century and potentially leads to the convergence of the day- and nighttime temperature sensitivity of ER (Figure 6). In addition, we assessed the influence of ER temperature sensitivity projections on ER. First, at the ecosystem scale, the DT-RH flux partitioning method with independent E_0,day_ and E_0,night_ accounts for 96% of the variation in monthly ER across all study sites, whereas DT accounts for 66% and NT accounts for 88% of that (Figure S4). Second, we used a new flux partitioning method (DT-RH) that estimated E_0,day_ and E_0,night_ separately and by coupling an automated machine learning (AutoML) model to predict ER for 2015 and 2100 (Figure 7), which explained approximately 85% of the variation in eddy covariance ER across global sites. Historical and future ER values are all highest in the tropical regions and lowest in cold and dry regions. However, we found an overestimation of ER at most tropical and temperate regions using current Earth system models compared to our ER estimates (DT-RH) in 2015 and 2010. Specifically, the variant DT-RH model estimates that ER releases 122.16 Pg C yr^−1^, 101.10 Pg C yr^−1^, and 116.77 Pg C yr^−1^ from the land to the atmosphere for 2015 and 2100 under SSP1-RCP2.6 and SSP5-RCP8.5, respectively, whereas that of CMIP6 models are 136.51 Pg C yr^−1^, 154.99 Pg C yr^−1^, and 210.98 Pg C yr^−1^, respectively.

## 3. Discussion

### 3.1. Higher Temperature Sensitivity of ER During the Day than at Night

The temperature sensitivity of ER is one of the most critical parameters in biogeochemical models for estimating total ER under climate change [24]. Our findings highlighted the dependence of ER temperature sensitivity at a temporal scale, showing that the temperature sensitivity of ER was lower during the day than at night across all biomes (Figure 2). This contrasts with the established notion of diurnal variation in ER as having a uniform temperature response [4,14,20], for which the temperature sensitivity of ER as a constant value for the whole year is only estimated using nighttime eddy covariance observations. The implications of these findings are substantial, as they challenge a classical assumption in many field studies, which underpins the extrapolation of semi-continuous empirical ER data obtained from eddy covariance [14] and closed chamber measurements [25] to daily scales.

Supported by previous studies reporting a diel hysteresis between respiration and temperature in forest [26] and grassland [27] systems, our study revealed a divergence in both parameters of the exponential model describing ER response to air temperature between nighttime and daytime. We attributed the higher E_0_ at night to the stronger temperature response of ER during nighttime, consistent with findings from previous studies [12,28]. Conceptually, the diurnal variation in ER is predominantly driven by the interactions between biotic (e.g., photosynthate availability) and abiotic (e.g., temperature, solar radiation, and moisture pulses) factors on the diel timescale [29,30,31]. One study [32] further demonstrated that diel phase lags between respiration and temperature can arise due to thermal and gas transport dynamics. During the day, temperature-independent respiration is mainly influenced by photosynthesis, which is widely recognized as the main driver of autotrophic respiration [33]. In contrast, nighttime respiration is primarily regulated by temperature [28]. This distinction emphasizes the different controls on daily ER, with photosynthesis exerting a stronger influence during the day and temperature becoming a more critical factor at night. Meanwhile, some studies highlighted that the autotrophic component of ER is likely to be more dynamic over the diel time due to the impact of photosynthesis and the greater temperature sensitivity of autotrophic respiration, whereas heterotrophic respiration is likely less to be dynamic as it experienced much smaller diel temperature fluctuations [34,35]. Thus, autotrophic respiration rather than heterotrophic respiration is the dominant component regulating the diel variation in ER and its temperature sensitivity [12,34,36].

In addition, our result is in consonance with some previous studies indicating that the temperature sensitivity of soil or leaf respiration was lower during the day than at night [15,16,37,38], but conflicts with other studies [12,18] indicating that temperature sensitivity of respiration was higher during the day than at night. A plausible explanation is that the different time scales between studies lead to the contradictory results, and some studies [12,18] only explored differences in respiration temperature sensitivity between daytime and nighttime from May to October. In our study, the day–night discrepancy in the temperature sensitivity of respiration from May to October was smaller than that noted in other months, and even some forest ecosystems showed slightly higher E_0,day_ than E_0,night_. One study [12] demonstrated that diel phase lags between respiration and temperature can result from varying contributions from heterotrophic and autotrophic respiration, and autotrophic respiration was the dominating component regulating the diel variation in the temperature sensitivity of ER. Thus, the higher E_0,day_ from May to October may be regulated by autotrophic respiration maxima occurring during warmer daytime conditions in the growing season.

### 3.2. Seasonal Variations in the Temperature Sensitivity of ER During the Day and Night

At a monthly scale, our results show that the day–night discrepancy E_0_ (ΔE_0_ = E_0,night_/E_0,day_) has strong seasonal variations for all biomes except for the seasonal variation noted for EBF and SAV, which was weak (Figure 3 and Figure 4). The diurnal difference in ER the temperature sensitivity between daytime and nighttime from June to September is smaller than that noted for other months. Specifically, E_0,day_ was slightly higher than E_0,night_ from June to September in forest and wetland ecosystems, which is in line with the results of [12,18], whereas E_0,day_ was significantly lower than E_0,night_ for other months. The seasonal heterogeneity of E_0_ during the day and night embodies the effect of various confounders on E_0_, which could involve multiple mechanisms.

First, the higher and relatively stable E_0,day_ from June to September can be attributed to elevated daytime temperature and leaf area across the Northern Hemisphere, which enhances soil organic matter (SOM) decomposition [39] and photosynthetic capacity, increasing total respiration rates, and consequently resulting in a higher E_0,day_ compared to other months. Meanwhile, summer temperatures typically fall within the optimal range for physiological vegetation processes [40], soil enzymatic functions, and microbial activities [41,42], which may contribute to the stability of E_0,day_ throughout the summer. Second, the lower apparent E_0,night_ between June and September than other months is in line with one study [16], and the effects may be limited by increasing temperature and decreasing soil moisture variability as well as shifts in photosynthate allocation during summer. Warming-associated soil drying can decrease respiration by promoting water limitations on microbial metabolism in dry or aerobic soils [43,44]. Also, the rate of phloem transport, a key determinant of lag time in below-ground allocation from photosynthate, is notably reduced in summer, as drought stress limits xylem translocation and delays the appearance of assimilates in the rhizosphere [43]. This seasonally induced lag in below-ground transport thus contributes to the pronounced disparity between E_0,day_ and E_0,night_ during summer.

Moreover, the seasonal variation magnitudes of E_0_ and ΔE_0_ vary greatly at different locations for the same biome (Figure 6 and Figure S2). Our analyses reveal a stronger seasonality of E_0_ and ΔE_0_ in colder climate regions, which is in line with the widely accepted assumption that E_0_ decreases with increasing temperature [29]. Meanwhile, compared to other terrestrial ecosystems, the lower seasonal variation in E_0_ for EBF and SAV may be attributed to the consistently higher temperature in tropical regions with lower seasonal variation in temperature, which may reduce seasonal differences in plant and microbial activity. In addition, the ASV of ΔE_0_ also showed a decrease with increasing temperature, implying that the temperature sensitivities of ER during the day and night may converge under future warming. This hypothesis is verified from the data shown in Figure 6. E_0,night_ indeed decreases under two future warming scenarios, whereas E_0,day_ slightly increases over tropical regions with future warming, suggesting that warming diminishes the day–night discrepancy in the temperature sensitivity of ER. This may due to thermal acclimation, which has been demonstrated to alter respiration responses to temperature [45,46]. Therefore, without accounting for the diel and seasonal variability of E_0_ or other confounding factors that impact E_0_, models would likely inaccurately estimate ER in response to climate change [9,16,29].

### 3.3. Implications for ER Predictions

Estimations of global ER directly affect the accuracy of ecosystem carbon budgets, which are crucial to make policy decisions to meet targets of environmental mitigation [13]. Our results suggest that ignoring differences in diurnal ER temperature sensitivity will have a profound effect on ER estimates. Also, we used fivefold cross-validation to assess the robustness of our results, which showed that DT-RH combined with AutoML models had high robustness for ER estimates and could account for 81%, 72%, and 92% of variations in ER at boreal, temperate, and tropical regions, respectively (Figure S6). We further compared the global spatiotemporal patterns of ER in 2015 and 2100 using DT-TH and CMIP6 products. The results suggest that the Earth system models of CMIP6 generally overestimate global ER, especially in 2100 under the SSP5-RCP8.5 scenario, where the estimates are approximately double those of ours. Moreover, the observed diel variations in basal E_0_ in our analysis contradicts the monotonic increase in respiration with increasing temperature predicted using current Earth system models, which challenges previous consensus and potentially leads to an overestimation of ER from sub-daily to annual scales if E_0,day_ is not considered. Therefore, to enhance projections of climate–carbon cycle feedback, Earth system models should be improved by incorporating the diel temperature response of respiration.

Generally, the temperature response functions used to model autotrophic respiration and heterotrophic respiration vary substantially between process-based models with most models using Q_10_, Arrhenius functions, or the equations of Lloyd and Taylor [8]. Nonetheless, numerous respiration models estimate daily respiration solely from daytime or nighttime measurements with a fixed temperature sensitivity of respiration [16,47,48]. However, ER is influenced by multiple biophysical factors, such as the time required for transporting photosynthetic products [49], heat and CO_2_ diffusion dynamics [32], and the interactive effects of temperature and moisture [50], which vary across different ecosystems. These heterogeneous conditions contribute to divergent diel patterns in ER and variations in its temperature sensitivity. Shifts in abiotic and biotic drivers might have contrasting effects on ER depending on the individual responses of autotrophic and heterotrophic respiration. For example, ref. [12] suggested that autotrophic respiration rather than heterotrophic respiration primarily regulated the diel variations in ER and its temperature response. Consequently, variations in the functions selected to simulate the temperature response of autotrophic and heterotrophic respiration may be the primary reason for the large differences in ER values predicted using the Earth system models.

In summary, understanding the temperature sensitivity of ER is crucial for accurately predicting the future land carbon sink under ongoing conditions of climate warming. Our work underscores that the conventional approach to deriving temperature sensitivity from long-term datasets may fail to capture short-term fluctuations in ER (e.g., hourly to daily), leading to stark bias [14,20]. By highlighting the substantial influence of E_0,day_ relative to E_0,night_ across diverse biomes, we emphasize the need for refined partitioning efforts in global carbon cycle models. Despite these important findings, most contemporary Earth system models do not account for the day–night discrepancy in the temperature response. Therefore, incorporating these insights can enhance our ability to accurately predict ecosystem CO_2_ emissions across various temporal scales, thereby advancing our understanding of climate feedback mechanisms and ecosystem carbon dynamics.

## 4. Materials and Methods

### 4.1. FLUXNET Data and Related Variables

We used eddy covariance carbon fluxes and meteorological data from the FLUXNET 2015 dataset (Tier 1; https://fluxnet.org/data/download-data/, accessed on 30 October 2022). The FLUXNET datasets were quality controlled, filtered, and gap-filled using consistent methods [51]. According to the International Geosphere-Biosphere Programme, the FLUXNET sites represent 9 distinct ecosystems: cropland (CRO), deciduous broadleaf forest (DBF), evergreen broadleaf forest (EBF), evergreen needleleaf forest (ENF), mixed forest (MF), grassland (GRA), savannas (SAV), shrubland (SHR), and wetland (WET). We used half-hourly or hourly carbon fluxes and their concurrent meteorological observations from 189 sites across the globe (Figure 1), including net ecosystem exchange (NEE_VUT_USTAR50), air temperature (Tair), nighttime air temperature (Tnight), daytime air temperature (Tday), solar radiation (SW_IN), precipitation (Pre), vapor pressure deficit (VPD), wind speed (WS), relative humidity (RH), and potential evapotranspiration (PET). For the missing RH value, we applied random forest (RF) method to fill the gap using T and VPD as drivers.

In addition to these flux and meteorological variables, the soil properties and leaf traits data for each site were extracted from global datasets according to its latitude and longitude. Soil organic carbon (SOC) and soil class were retrieved from the Regridded Harmonized World Soil Database v.1.2 from the Oak Ridge National Laboratory Distributed Active Archive Center for Biogeochemical Dynamics (https://daac.ornl.gov/SOILS/guides/HWSD.html, accessed on 15 November 2022). Surface volumetric soil moisture (0–7cm, SWC) data were accessed from the fifth-generation ECMWF atmospheric reanalysis of the global climate (ERA5). Leaf nitrogen concentration (LNC), leaf phosphorus concentration (LPC), specific leaf area (SLA), and leaf dry matter content (LDMC) were extracted from https://isp.uv.es/code/try.html?tdsourcetag=s_pctim_aiomsg (accessed on 15 November 2022). Solar-induced chlorophyll fluorescence (SIF) [52] was collected from https://figshare.com/articles/dataset/CSIF/6387494 (accessed on 20 November 2022).

To compute the global products at yearly resolution via upscaling, we also extracted gridded monthly daily mean temperature (Tair), average daily minimum (Tmin) and maximum temperature (Tmax), precipitation, and wind speed time series data at 0.5° spatial resolution. These data were obtained from Climate Research Unit (CRU TS v. 4.04, https://crudata.uea.ac.uk/cru/data/hrg/, accessed on 15 November 2022). We collected monthly surface solar radiation (Rg) and air relative humidity (RH) data from the fifth-generation ECMWF atmospheric reanalysis of the global climate (ERA5) at 0.5° spatial resolution. We collected global standardized precipitation ET index (SPEI) data with 0.5° spatial resolution and a monthly time resolution as a measure of drought intensity (https://spei.csic.es/database.html, accessed on 20 December 2022). We obtained the monthly leaf area index (LAI) dataset with a spatial resolution of 0.5° from the Global Land Surface Satellite products (http://www.glass.umd.edu/index.html, accessed on 10 March 2023). We obtained the biweekly Global Inventory Modeling and Mapping Studies third-generation (GIMMS-3g) NDVI dataset with a spatial resolution of 1/12° (https://iridl.ldeo.columbia.edu/SOURCES/.NASA/.ARC/.ECOCAST/.GIMMS/.NDVI3g/.v1p0/index.html?Set-Language=en, accessed on 15 November 2022). Here, we composited the biweekly GIMMS-NDVI3g data to monthly temporal resolution by selecting the higher of the two composites in the same month. Additionally, the datasets mentioned above for two future corresponding shared socioeconomic pathway-representative concentration pathway (SSP-RCP) scenarios were collected from the Coupled Model Intercomparison Project models (CMIP6) (https://aims2.llnl.gov/search/cmip6/, accessed on 25 May 2024). Moreover, the three model simulations from CMIP6 were considered in our analysis, including BCC-CSM2-MR [53], CanESM5 [54], and CMCC-ESM2 [55]. From these model outputs, we extracted years 2015 and 2100 for comparison with our inferred estimates of ER.

### 4.2. Modified Flux Partitioning Method (DT-RH)

The DT-RH flux partitioning method has been successfully applied and demonstrated at three sites [13]. Here, we briefly recapitulate the method. The DT-RH modeling strategy is based on the following assumptions: (1) the temperature sensitivity of ER is different between daytime and nighttime; (2) the effect of moister conditions on ER varies from negative in wet ecosystems to positive in dry ones. This method was modified from [20] by adding a relative humidity (RH) term as follows:

(1) $$\mathrm{NEE}=\frac{\alpha \beta Q}{\alpha Q+\beta }+R_{ref}\exp(E_{0}(\frac{1}{T_{ref}-T_{0}}-\frac{1}{T_{air}-T_{0}}))\times f\left(h\right),$$

(2) $$f\left(h\right)=1-\varepsilon \times \frac{\mathrm{RH}-\bar{\mathrm{RH}}}{\bar{\mathrm{RH}}},$$

(3) $$\beta =\{\begin{array}{c} \beta_{0}\exp\left(-k\left(VPD-VPD_{0}\right)\right),VPD>VPD_{0},\\ \beta =\beta_{0},VPD<VPD_{0} \end{array}\;$$

where *α* (μmol C J^−1^) is the canopy-scale quantum yield, which represents the initial slope of the light–response curve; and *β* (μmol C m^−2^ s^−1^) is the maximum CO_2_ uptake rate of the canopy at light saturation, which in turn is regulated by *VPD*. The threshold of *VPD* (*VPD*_0_) is set to 10 hPa. *k* is a coefficient used to quantify the response of the maximum CO_2_ uptake to *VPD*, and *Q* is the global radiation (W m^−2^). *R_ref_* (μmol C m^−2^ s^−1^) is the base respiration rate at the reference temperature (*T_ref_* = 15 °C). *E*_0_ (K) is the temperature sensitivity of ER, and *T_air_* (°C) is the air temperature. *T*_0_ (°C) is kept constant at −46.02 °C. $\bar{\mathrm{RH}}$ is the mean value of RH at a certain data window, and ε is a fitted site-specific parameter, which indicates the positive and negative relationship between $\bar{\mathrm{RH}}$ and the standardized respiration rate (i.e., the ratio of daytime ER (DER) and R_ref,day_, DER/R_ref,day_, referred to as site characteristics) across different sites. The standardized respiration rate (DER/R_ref,day_) was first calculated from the DT-*E*_0_ method [13]. The parameter *ε* was set to −1 if a negative correlation existed between DER/R_ref,day_ and $\bar{\mathrm{RH}}$; otherwise, it was set to 1. The other parameters (*α, β*_0_, *k*, daytime *R_ref_* (R_ref,day_), and *E*_0_ (E_0,day_)) were estimated by fitting the entire model to the daytime data, whereas nighttime *R_ref_* (R_ref,night_) and *E*_0_ (E_0,night_) were estimated using nighttime data. We then applied R_ref,day_ and E_0,day_ to estimate daytime ER and used R_ref,night_ and E_0,night_ to estimate nighttime ER.

### 4.3. Machine Learning Model Training and Validation

We used machine learning methods (i.e., random forest (RF) [56] and automated machine learning (AutoML) [57]) based on input datasets for the global meteorological data and remote sensing data to generate the global estimates of E_0,day_, E_0,night_, and ER for the years 2015 and 2100, separately. The RF algorithm is well equipped to address the complexity of ecological data due to its ensemble nature, which enables the integration of multiple decision trees to capture intricate interactions among variables. Compared to traditional manual approaches for feature selection and hyperparameter optimization, the application of AutoML significantly enhances computational efficiency and reduces the subjectivity and potential biases inherent in manual processes [58]. AutoML is adept at autonomously selecting the optimal combination of features and determining the best-performing prediction model during hyperparameter training, which notably reduces the risk of overfitting [58,59]. This approach not only streamlines the modeling workflow but also ensures a more objective and robust analytical framework. The current version of AutoML (in H_2_O 3.16) trains and cross-validates the generalized linear models (GLMs), a default random forest, an extremely randomized forest, a random grid of gradient boosting machines (GBMs), and a random grid of deep neural nets. Then, it trains two stacked ensemble models at the end. The ensembles consist of either all base models or only the best-performing base models from each model family. H_2_O AutoML then ranks the performance of individual models and model ensembles using an internal cross-validation (CV). The best-performing model is used for prediction [59]. In this study, we use the ensemble that contains all the models to estimate target variable.

For day- and nighttime *E*_0_ estimates in 2015, we first convert monthly *E*_0_ data to a yearly scale at each site, yielding a global dataset composed of yearly estimates of local E_0_ and forcing data (i.e., latitude, longitude, Tair, Tmin, Tmax, MAP, PAR, SPEI, RH, WS, and NDVI) from each tower. Second, we used the global dataset to train the machine learning models and then created maps of gridded yearly E_0,day_ and E_0,night_ at 0.5° × 0.5° resolution. We used the same steps to estimate day- and nighttime *E*_0_ in 2100 and ER in 2015 and 2100, except for replacing the forcing data with latitude, longitude, Tair, Tmin, Tmax, MAP, PAR, RH, WS, and LAI.

A fivefold cross-validation was used to evaluate the performance of the two machine learning models for annual E_0_ and ER estimations. During each model fitting, one of the partitions was reserved for validation, while the other four were used for training. The random group splitting was stratified based on ecosystem types and climate zones to ensure coverage of the full range of ecosystem types and climate zones in both training and testing data. Meanwhile, each flux site was exclusively allocated to a group to assess the performance of the model in unknown locations. This modeling process was repeated 10 times, and the performance metrics, including the coefficient of determination (R^2^) and the root mean squared error (RMSE), were averaged to describe the final performance. Additionally, to quantify the uncertainty of machine learning models, we trained an ensemble of 10 machine learning models using bootstrapped samples and generated an ensemble of 10 E_0_/ER values. Each bootstrapped sample set contained all ecosystem types and climate zones. The coefficient of variance was shown in Figure S5. Overall, the RF model explains approximately 60%~84% of the variation in E_0,day_ and E_0,night_ estimates, exhibiting better performance than that of the AutoML model (Table S1) but with small performance differences. Conversely, the performance of the AutoML model was more robust in predicting ER than the RF model (Table S2). However, the differences between models are challenging to explain, as the reasons for the models’ results are obscured by their black box character. We attribute the differences in model prediction accuracy to variations in target variables. Specifically, E_0_, as a model parameter and a relative metric with a more constrained range [20], requires consistency with the training data to ensure the scientific validity of the results. In contrast, the range of total ecosystem respiration under future climate scenarios is not fixed. While the RF model demonstrates strong predictive performance within the bounds of the training data, its ability to extrapolate beyond this range may be limited. AutoML, however, offers greater flexibility in addressing future scenario predictions by leveraging a combination of algorithms and automated hyperparameter tuning.

### 4.4. Statistical Analysis

We applied the Markov chain Monte Carlo method [60] to parameterize E_0_ during the day and night in the DT-RH model every 7 days with a 5-day moving window at each site. Detailed methods are described in [13]. Then, we average the 7-day E_0,day_ and E_0,night_ values to the monthly scale. An independent sample t-test was used to analyze the significance of the difference between daytime and nighttime E_0_ at nine ecosystems, separately. The seasonal variations in E_0_ were calculated as the ratio of the standard deviation of E_0_ to its monthly mean value each year at each site. For each year, the seasonal amplitude (ASV) in the day–night discrepancy in ER temperature sensitivity (ΔE_0,ASV_) was calculated as the difference between highest and lowest monthly E_0,night_/E_0,day_ ratios from January to December. The relative contributions of climate, soil, and plant variables to ΔE_0,ASV_ were explored using a principal component analysis (PCA) and general linear models. Meanwhile, the relative importance of the various drivers in regulating E_0_ variation was examined using a random forest model. Meanwhile, we additionally quantified the relative contributions of climate, plant, and soil factors in regulating E_0_ variations based on the RF model. We defined three regions [61] based on annual air temperature (MAT): boreal (MAT < 2 °C), temperate (2 °C ≤ MAT ≤ 17 °C), and tropical (MAT > 17 °C).

## Figures and Tables

**Table 1:** General linear models predicting the effects of environmental and plant variables on ΔE_0,ASV_.

Model Rank	AICc	R^2^	*p*-Value	Parameter (±SE)	
				Dim 1	Dim 2
1	359.15	0.38	<0.001	0.14 (0.02)	−0.14 (0.02)
2	394.91	0.23	<0.001	0.14 (0.02)	
3	410.98	0.15	<0.001		−0.14 (0.03)

AICc: second-order Akaike information criterion.

## Data Availability

All data are available within the manuscript, and further information can be obtained upon request from the corresponding author.

## Supplementary Materials

The following supporting information can be downloaded at: https://www.mdpi.com/article/10.3390/plants13233321/s1, Figure S1: Seasonal variations in the day–night temperature sensitivity of ER (E_0,night_, E_0,day_ and ΔE_0_) at nine ecosystems; Figure S2: Seasonal variation in the temperature sensitivity of ER during the night (E_0,night_, a) and day (E_0,day_, b) and the day–night discrepancy in the temperature sensitivity of ER (ΔE_0_, c) with latitude and ecosystem types; Figure S3: Relative importance of climate and plant variables on the temporal variation in ER temperature sensitivity during nighttime (E_0,night_, a) and daytime (E_0,day_, b); Figure S4: Model validations of ER estimates across all study sites using DT-RH (a), DT (b), and NT (c) flux partitioning methods; Figure S5: The variance coefficient of machine learning models on E_0,night_/E_0,day_/ER spatiotemporal estimations around the globe; Figure S6: Model performance evaluation of the automated machine learning (AutoML) model for ER estimates across zones and biomes based on fivefold cross-validation; Table S1: Summary of the model performance evaluation of machine learning models for E0 estimates (i.e., random forest (RF) and automated machine learning (AutoML)) based on fivefold cross-validation; Table S2: Summary of the model performance evaluation of machine learning models for ER estimates (i.e., random forest (RF) and automated machine learning (AutoML)) based on fivefold cross-validation.
