# Supplementary material for: Warming Diminishes the Day–Night Discrepancy in the Apparent Temperature Sensitivity of Ecosystem Respiration
Source: Plants (Basel). 2024 Nov 26;13(23):3321. doi: 10.3390/plants13233321 (PMC11644270; doi:10.3390/plants13233321)
Supplement: Supplementary file 1 [file plants-13-03321-s001.zip › plants-3310410-supplementary.pdf]

Supporting Information

Table S1 Summary of the model performance evaluation of machine learning models for E_0 estimates (i.e., random forest (RF) and automated machine learning (AutoML)) based on five-fold cross-validation. R^2: the coefficient of determination; RMSE: the root mean squared error.

**Model evaluation statistics**	**RF**				**AutoML**			
	2015/2100$E_{0,night}$		2015/2100$E_{0,day}$		2015/2100$E_{0,night}$		2015/2100$E_{0,day}$	
	R^2	RMSE	R^2	RMSE	R^2	RMSE	R^2	RMSE
Training dataset	0.61	35.36	0.86	16.26	0.82	20.36	0.96	7.89
Testing dataset	0.59	33.85	0.84	16.38	0.57	34.42	0.83	16.38

Table S2 Summary of the model performance evaluation of machine learning models for ER estimates (i.e., random forest (RF) and automated machine learning (AutoML)) based on five-fold cross-validation. R^2: the coefficient of determination; RMSE: the root mean squared error.

**Model evaluation statistics**	**RF**		**AutoML**	
	2015/2100ER		2015/2100ER	
	R^2	RMSE	R^2	RMSE
Training dataset	0.83	14.70	0.98	5.13
Testing dataset	0.80	15.33	0.85	14.72

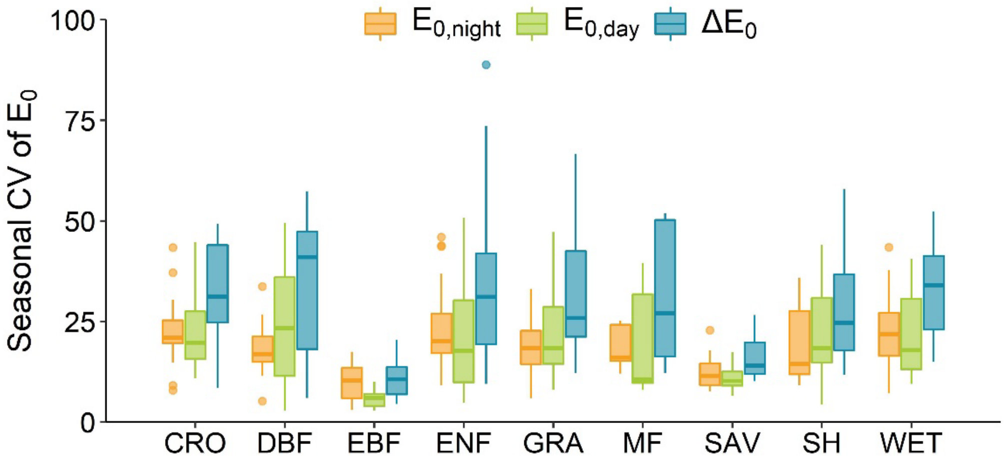

Figure S1 Seasonal variations in the day－night temperature sensitivity of ER ($E_{0,night}$, $E_{0,day}$ and ΔE_0) at nine ecosystems, including cropland (CRO), deciduous broadleaf forest (DBF), evergreen broadleaf forest (EBF), evergreen needleleaf forest (ENF), mixed forest (MF), grassland (GRA), savannas (SAV), shrubland (SHR), wetland (WET).

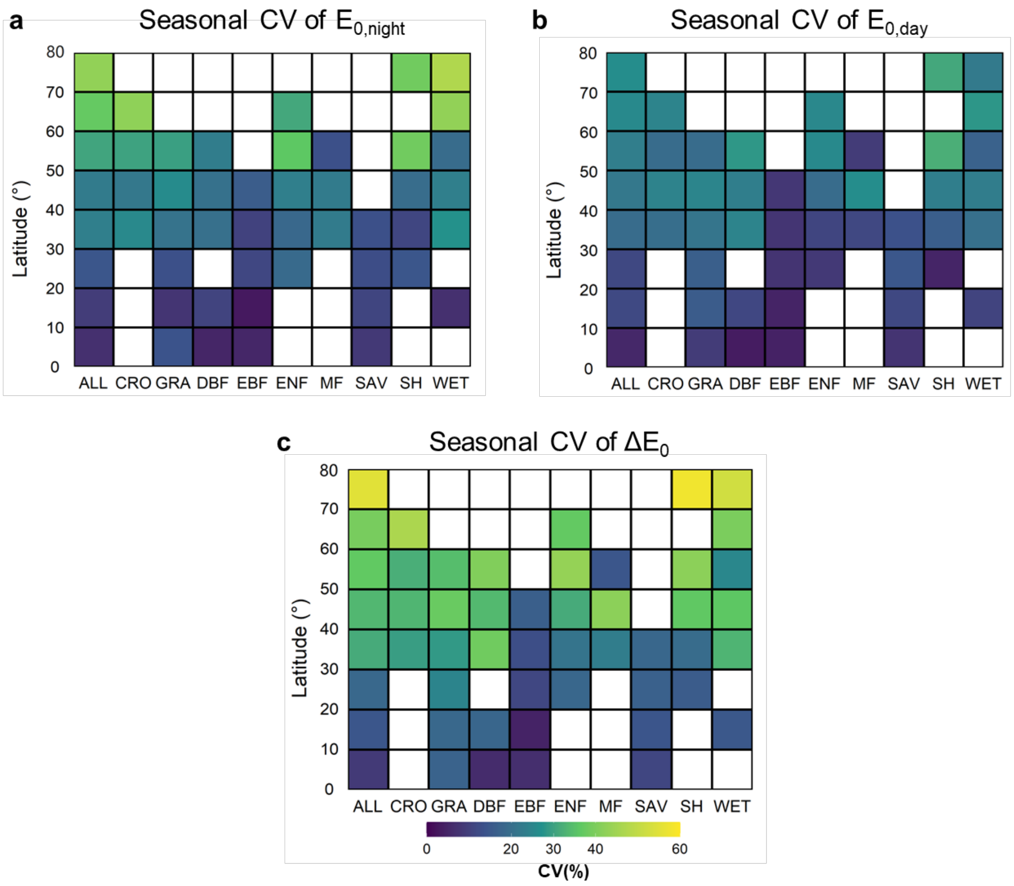

Figure S2 Seasonal variation in the temperature sensitivity of ER during the night ($E_{0,night}$, a) and day ($E_{0,day}$, b) and the day–night discrepancy in the temperature sensitivity of ER (ΔE_0, c) with latitude and ecosystem types. CRO, cropland; DBF, deciduous broadleaf forest; EBF, evergreen broadleaf forest; ENF, evergreen needleleaf forest; MF, mixed forest; GRA, grassland; SAV, savannas; SHR, shrubland; WET, wetland; CV, coefficient of variation.

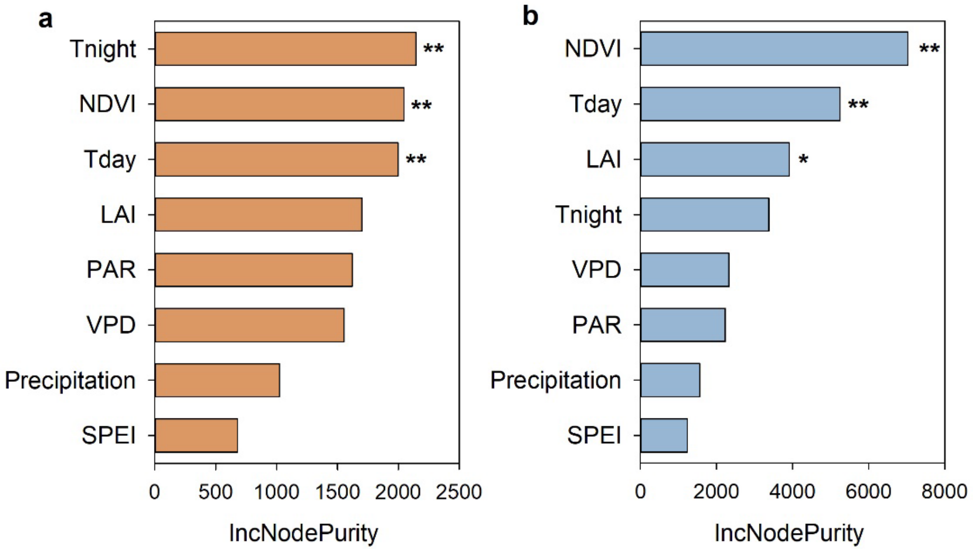

Figure S3 Relative importance of climate and plant variables on the temporal variation in ER temperature sensitivity during nighttime ($E_{0,night}$, a) and daytime ($E_{0,day}$, b). *$p < 0.05$, **$p < 0.01$. Tnight, nighttime air temperature; Tday, daytime air temperature; PAR, solar radiation; VPD, vapour pressure deficit; SPEI, standardized precipitation-ET index; LAI, leaf area index; NDVI, normalized difference vegetation index;

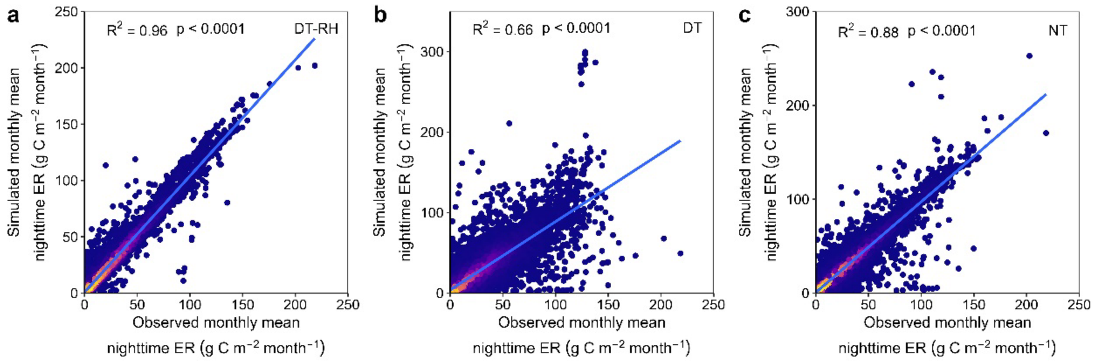

Figure S4 Model validations of ER estimates across all study sites using DT-RH (a), DT (b), and NT (c) flux partitioning methods. DT-RH, daytime method with air relative humidity; DT, daytime method; NT, nighttime method.

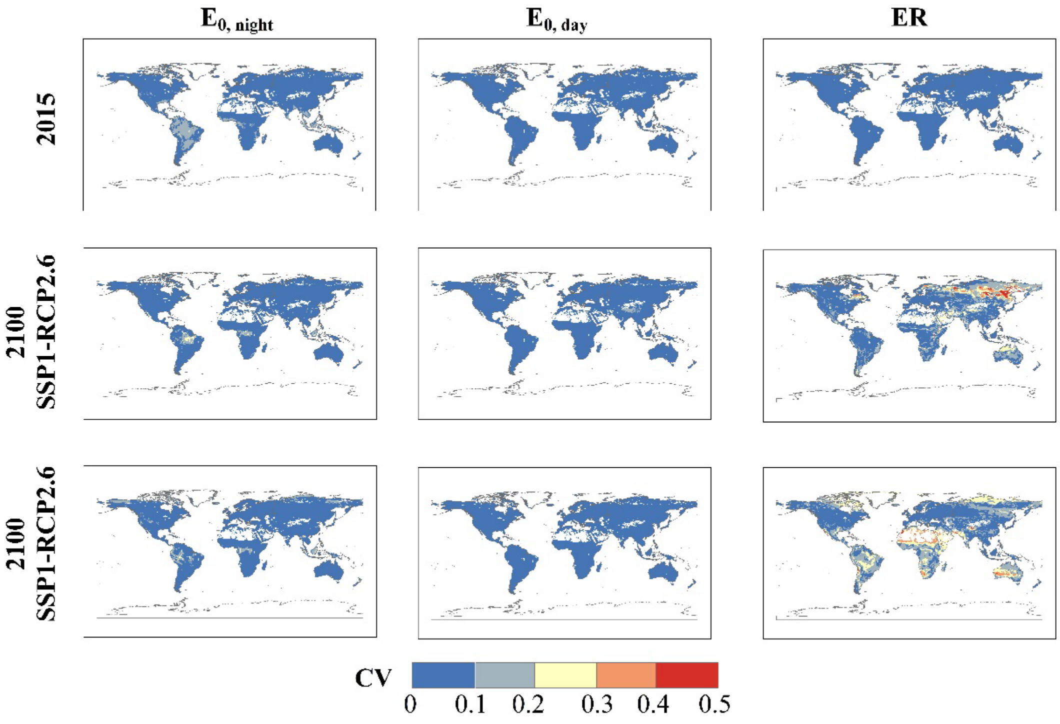

Fig. S5 The variance coefficient of machine learning models on $E_{0,night}/E_{0,day}$/ER spatiotemporal estimations around the globe.

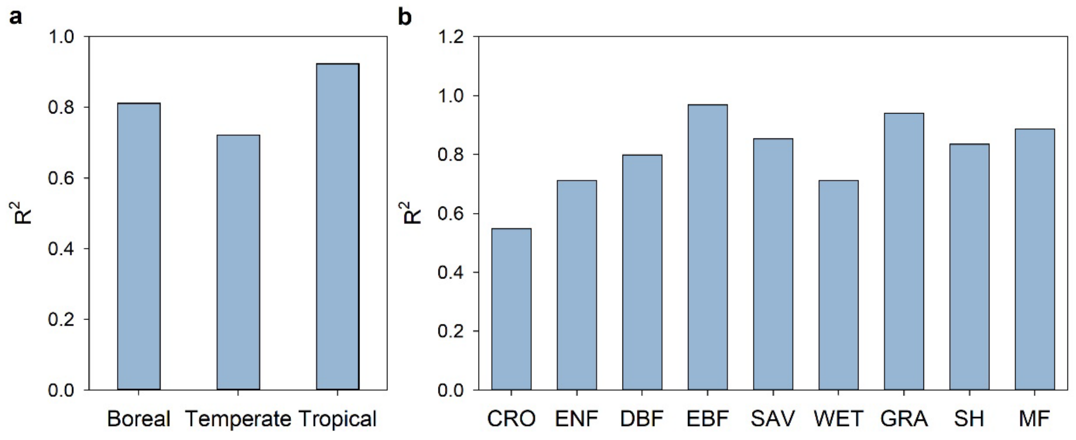

Figure S6 Model performance evaluation of the automated machine learning (AutoML) model for ER estimates across zones and biomes based on five-fold cross-validation. R^2: the coefficient of determination. CRO, cropland; DBF, deciduous broadleaf forest; EBF, evergreen broadleaf forest; ENF, evergreen needleleaf forest; MF, mixed forest; GRA, grassland; SAV, savannas; SH, shrubland; WET, wetland.
